# Whole-mount single molecule FISH method for zebrafish embryo

**DOI:** 10.1038/srep08571

**Published:** 2015-02-25

**Authors:** Yuma Oka, Thomas N. Sato

**Affiliations:** 1 Kyoto University, Graduate School of Biostudies, Kyoto, Japan; 2 ERATO Sato Live Bio-Forecasting Project, Japan Science and Technology Agency (JST), Kyoto, Japan; 3 Advanced Telecommunications Research Institute International (ATR), The Thomas N. Sato BioMEC-X Laboratories, Kyoto, Japan; 4 Nara Institute of Science and Technology, Graduate School of Biological Sciences, Nara, Japan; 5 Cornell University, Department of Biomedical Science, NY, USA; 6 Centenary Institute, Sydney, Australia

## Abstract

Noise in gene expression renders cells more adaptable to changing environment by imposing phenotypic and functional heterogeneity on genetically identical individual cells. Hence, quantitative measurement of noise in gene expression is essential for the study of biological processes in cells. Currently, there are two complementary methods for quantitatively measuring noise in gene expression at the single cell level: single molecule FISH (smFISH) and single cell qRT-PCR (or single cell RNA-seq). While smFISH has been developed for culture cells, tissue sections and whole-mount invertebrate organisms, the method has not been reported for whole-mount vertebrate organisms. Here, we report an smFISH method that is suitable for whole-mount zebrafish embryo, a popular vertebrate model organism for the studies of development, physiology and disease. We show the detection of individual transcripts for several cell-type specific and ubiquitously expressed genes at the single cell level in whole-mount zebrafish embryo. We also demonstrate that the method can be adapted to detect two different genes in individual cells simultaneously. The whole-mount smFISH method described in this report is expected to facilitate the study of noise in gene expression and its role in zebrafish, a vertebrate animal model relevant to human biology.

Cell-to-cell variability in gene expression is inherent in all living cells^1^,^2^,^3^,^4^. Such stochastic gene expression imposes phenotypic and functional variations on individual cells and appears to facilitate their adaptability to varying environmental conditions^5^,^6^. The quantitative measurements of gene expression in individual cells are conventionally performed by two methods: single-cell qRT-PCR (or RNA-seq)^7^,^8^,^9^,^10^,^11^,^12^ and single molecule fluorescent *in situ* hybridization (smFISH)^12^,^13^,^14^,^15^,^16^. Single-cell qRT-PCR (or RNA-seq) can be applied to measure the transcript level at the single cell level as long as each cell can be sorted and individually harvested. In contrast, smFISH does not require harvesting individual cells, thus allowing quantitative determination of the transcript level in each individual cell *in situ*. Protocols for smFISH were originally developed with single cell organisms in culture such as yeast and mammalian cells^13^,^15^,^16^. These protocols were later adapted for multicellular invertebrate organisms such as fly and worm^13^,^17^,^18^,^19^, providing useful and important information of gene expression noise with spatial resolution at the whole organismal level. Subsequently, it was also applied to histological sections of mouse^20^, thus providing some data on gene expression noise with spatial resolution in a vertebrate animal. However, as the method was only applicable to histological sections, the obtainable data are useful but limited in spatial resolution. Furthermore, it is labor-intensive to prepare and process hundreds of sections for smFISH. In order to obtain data on gene expression noise in individual cells with spatial resolution but without preparing hundreds of sections, a protocol applicable to a whole-mount vertebrate animal is desirable. Hence, we developed and report here an smFISH protocol suitable for whole-mount zebrafish embryo, a commonly used vertebrate animal model for the studies of development, physiology and disease^21^.

## Results

We initially applied the established smFISH protocol for mouse culture cells to whole-mount zebrafish embryos^16^, but it resulted in low signal-to-noise ratio, failing to yield any distinguishable dot-like signals of individual transcripts (Supplementary Figure S1, the right panel). Therefore, we modified the protocol, in particular, prehybridization treatment steps by referring to conventional *in situ* hybridization (i.e., non-smFISH *in situ* hybridization) protocols for whole-mount zebrafish embryos. In the conventional *in situ* hybridization protocol, a methanol pretreatment step is always included prior to prehybridization steps. In contrast, no such pretreatment steps are included in the smFISH protocol for mouse culture cells^16^. Therefore, we included a methanol pretreatment step and found that such a step is absolutely critical, yielding distinguishable fluorescent dot signals that presumably represent individual transcripts (Supplementary Figure S1).

With this and other modifications such as fixation and washing conditions, we established an smFISH protocol suitable for whole mount zebrafish embryo (see Methods), with which we succeeded in detecting dot signals. We then examined whether the number of fluorescent dots quantitatively reflects the transcript level in individual cells. For this purpose, 12 hours post fertilization (hpf) *Tg(olig2:egfp)* transgenic zebrafish embryos, in which EGFP is expressed in progenitors for motor neurons (pMNs) of neural plate, were used. To detect and visualize individual *egfp* transcripts, a set of thirty-two 20-base DNA oligonucleotides against *egfp* individually labeled with carboxytetramethylrhodamine (TAMRA) at the 3′ end was used as probes.

**Figure 1:**
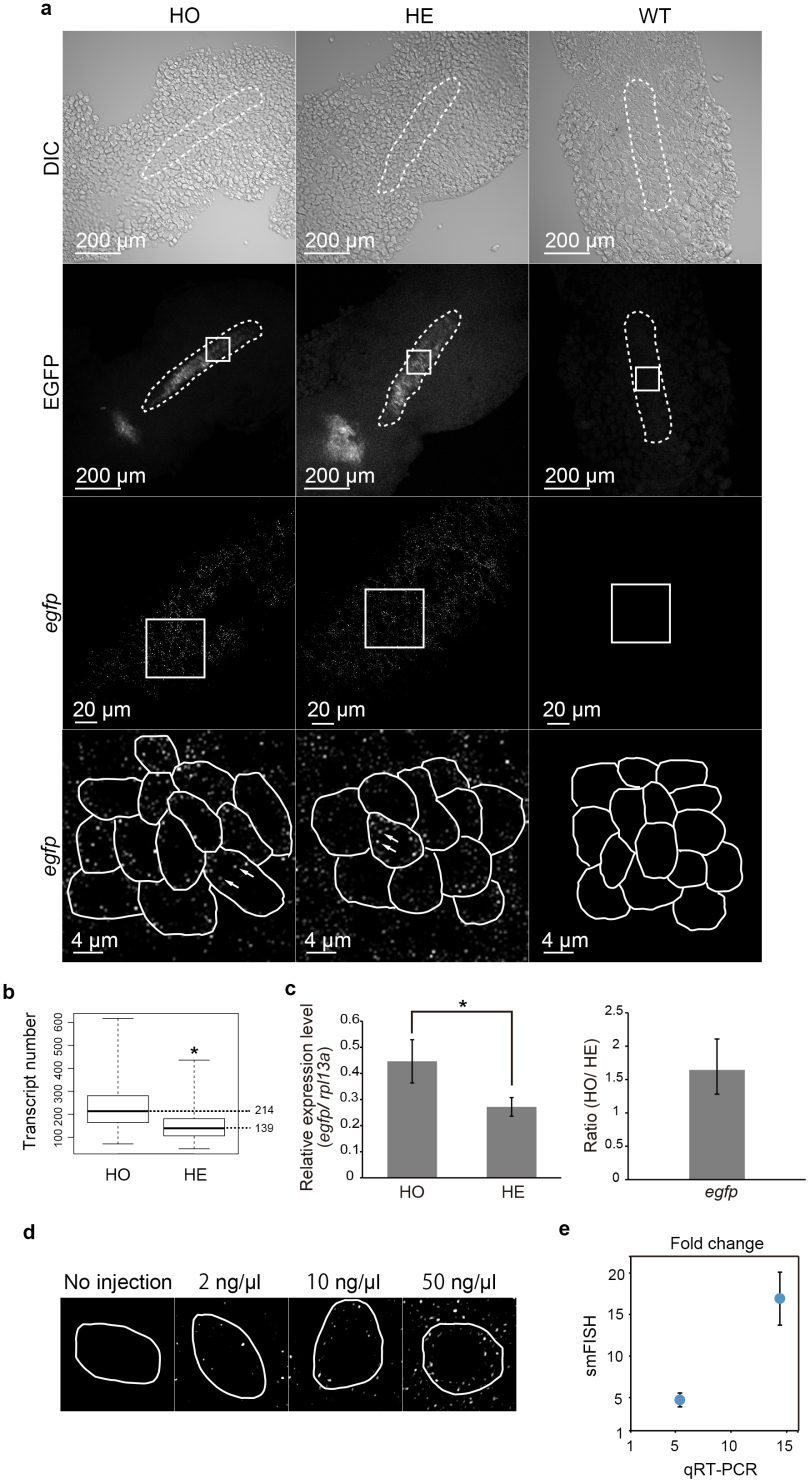
Validation of whole-mount smFISH protocol using a transgenic reporter line. (a) Results for homozygous (HO) and hemizygous (HE) *Tg(olig2:egfp)* transgenic and wild type (WT) zebrafish embryos are shown. The intact embryo was imaged by confocal microscopy. Shown are images of DIC (top row), EGFP fluorescence (second row) and the images of hybridization with the probe set for *egfp* conjugated with TAMRA (third and bottom rows). The smFISH hybridization images (third row) are those boxed in the EGFP fluorescence images (second row). Higher magnifications (bottom row) are filtered and threshold applied single plane fluorescent confocal images and correspond to the boxed areas in the lower magnification hybridization images (second and third rows). Individual cells are outlined by white enclosing lines in the higher magnification of the hybridization images and representative dot-like signals are indicated by arrows (bottom row). (b) Box plots showing the *egfp* transcript number. *p = 2.5e-16 (t-test). Mean values for each genotype are indicated on the right. (c) Quantitative real-time PCR for *egfp* in homozygous (HO) and hemizygous (HE) *Tg(olig2:egfp)* embryos. The level of *egfp* transcripts was normalized to those of *rpl13a* and shown as relative expression level. *p = 0.001081 (t-test). Data are shown as mean ± s.d. (d) Single plane fluorescent confocal images, each showing a representative cell from an uninjected and an embryo injected with 1nl of 2 ng/μl, 10 ng/μl, 50 ng/μl of *in vitro* transcribed *egfp* RNA. Individual cells are indicated by white enclosing lines. (e) A graph showing the linear correlation between the fold increases of the injected *egfp* RNA present in individual cells as determined by qRT-PCR and the fold increases of the number of fluorescent dots in individual cells.

Fluorescent dot signals, each presumably representing individual *egfp* transcripts, are specifically detected in pMNs of neural plate cells, but not in any of the EGFP-negative cells outside neural plate, of 12 hpf whole-mount *Tg(olig2:egfp)* zebrafish embryos (Figure 1a, left and middle column panels). No signals are detected in any cells of wild type zebrafish embryos (Figure 1a, right column panels). Furthermore, the increase in the number of fluorescent dots in homozygous transgenic embryo (HO) (the mean transcript number: 214) as compared to those in hemizygous transgenic embryo (HE) (the mean transcript number: 139) quantitatively matches to the increase in the expression level measured by qRT-PCR (1.6-fold increase) (Figure 1b, c, Supplementary Figure S2).

In addition, known amounts of *in vitro* transcribed *egfp* RNA were injected into wild type zebrafish embryos which were then subjected to the whole-mount smFISH and the number of fluorescent dots were counted (Figure 1d, e). The amounts of the injected *egfp* RNA present in the embryos were confirmed by qRT-PCR (Figure 1e). This result shows the number of dots increases linearly according to the increasing amounts of the injected *egfp* RNA present in individual embryos (Figure 1d, e). These results demonstrate the specificity of the fluorescent dot signals and also suggest that each fluorescent dot represents single *egfp* transgene transcript molecule.

The notion that each dot represents a single transcript molecule was further investigated by measuring the distribution of individual dot intensity in individual cells (Supplementary Figure S3). The result indicates that nearly 90%–100% of the dots show very similar fluorescent intensity (0.25–0.35). Only 2 dots out of a total of 503 dots (4 cells combined) exhibit intensity that is 2-fold (i.e. approximately 0.5) of the main intensity peak (0.25–0.3), suggesting that only about 0.4% of the fluorescent dots may be conglomerates of the transcripts. Taken all together, each fluorescent dot is likely to represent a single *egfp* transcript molecule.

**Figure 2:**
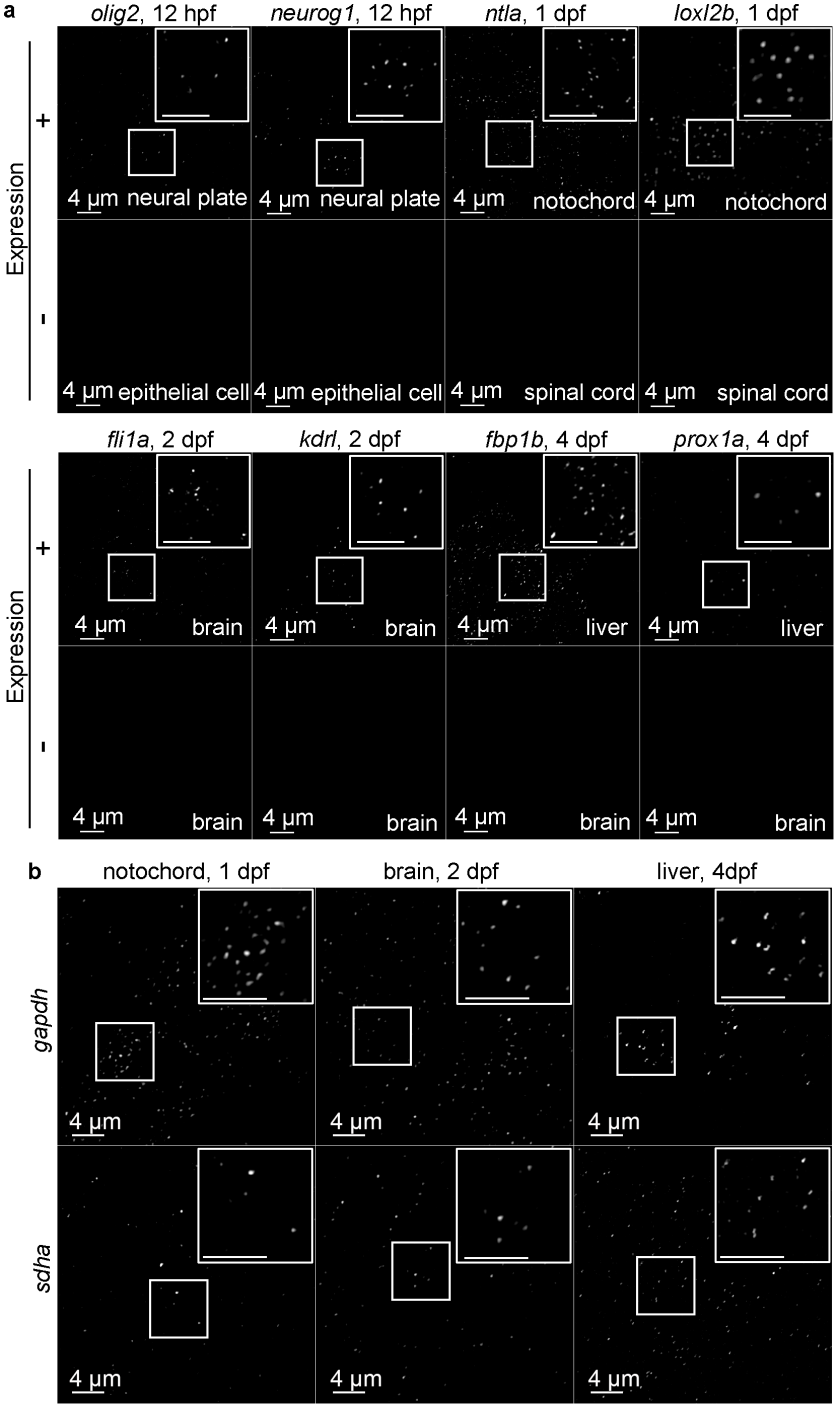
Whole-mount smFISH for endogenous genes in organs/tissues. (a) Filtered and threshold applied single plane confocal images of the indicated tissues of embryos subjected to the whole-mount smFISH for endogenous genes specifically expressed in the organs/tissues. The boxed areas are shown as higher magnification at the right top corner in each panel (top rows). The organs/tissues where the genes are known not to be expressed are shown as negative control (bottom rows). (b) Filtered and threshold applied single plane confocal images of the indicated tissues of embryos subjected to the whole-mount smFISH protocol for ubiquitously expressed genes. The boxed areas are shown as higher magnifications at the right top corner in each panel. In both (a) and (b), shown are confocal images taken with intact whole-mount embryos, except the panels labeled as “brain” that are taken with the head tissue separated from the rest of the body.

Next, we examined whether the protocol works for endogenous genes. Probes specific to several genes that show preferential expression in some cell types and organs were tested (see Supplementary Table S1 for the list of the genes examined). The expression of *olig2*^22^,^23^ and *neurog1*^22^ is detected as dots in neural plate cells at 12 hpf, where the expression of these two genes are expected (Figure 2a). In contrast, no signals are detected in epithelial cells at the same stage, where they are not supposed to be expressed (Figure 2a). The expression signals for *ntla*^24^ and *loxl2b*^25^ are detectable in notochord, but not in spinal cord at 1 dpf (Figure 2a). The expression of vascular endothelial genes, *fli1a*^26^ and *kdrl*^27^ are detected in some cells in brain at 2 dpf, but not in other cells (Figure 2a). Fluorescent dots for both *fbp1b* (http://zfin.org/cgi-bin/webdriver?MIval=aa-imageview.apg&image_table=image&OID=ZDB-IMAGE-021210-627)^28^ and *prox1a*^29^ are detected in liver at 4 dpf, but not in brain (Figure 2a). These preferential expression patterns of the genes are in agreement with their known expected expression patterns. We also tested probes for two relatively ubiquitously expressed genes, *gapdh* and *sdha* (Figure 2b, Supplementary Table S1). The typical fluorescent dots using these probes are detectable in multiple tissues/organs as expected (Figure 2b, Supplementary Table S1). We used two different fluorescent dyes, TAMRA and Quasar 670, for each probe, and found that all probes work with TAMRA, but some fail with Quasar 670 (Supplementary Table S1).

**Figure 3:**
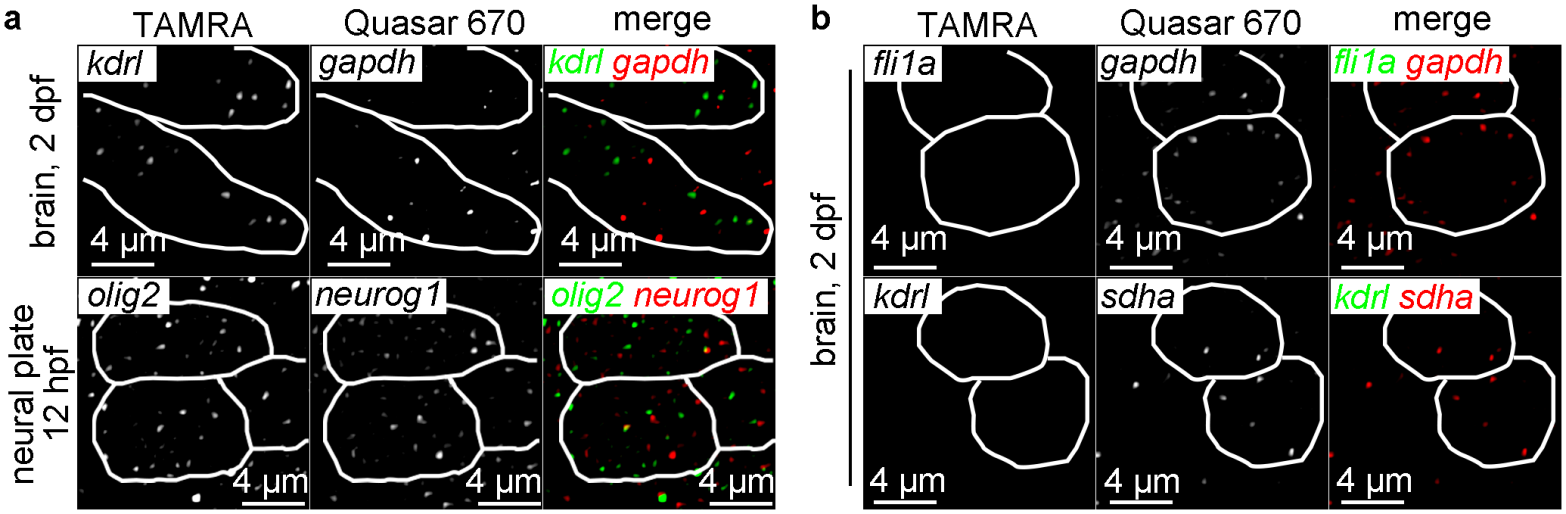
Expression analysis of two different genes using whole-mount smFISH protocol. (a) Filtered and threshold applied single plane confocal images of the indicated tissues of embryos subjected to the whole-mount smFISH protocol showing the co-localization of two genes (*kdrl* and *gapdh, olig2* and *neurog1*) in the same single cells (outlined by white lines). (b) Filtered and threshold applied single plane images of brain (2dpf) of embryos subjected to the whole-mount smFISH protocol showing individual cells (outlined by while lines) that express one ubiquitously expressed gene (*gapdh*, *sdha*), but not cell type-specific (endothelial cell-specific) genes (*fli1a*, *kdrl*). In both (a) and (b), shown are confocal images taken with intact whole-mount embryos, except the panels labeled as “brain” that are taken with the head tissue separated from the rest of the body.

We next examined whether the expression of two different genes can be simultaneously detected by our smFISH protocol. Using our protocol, two different genes (*kdrl* and *gapdh*, *olig2* and *neurog1*) can be detected in the same cell as expected (Figure 3a). In brain, some cells that express ubiquitously expressed genes (*gapdh*, *sdha*), but not endothelial genes (*fli1a*, *kdrl*) are also found (Figure 3b). This result serves to show that non-endothelial cells in brain can be positive for ubiquitously expressed genes (*gapdh*, *sdha*) while not expressing endothelial genes (*fli1a*, *kdrl*).

**Figure 4:**
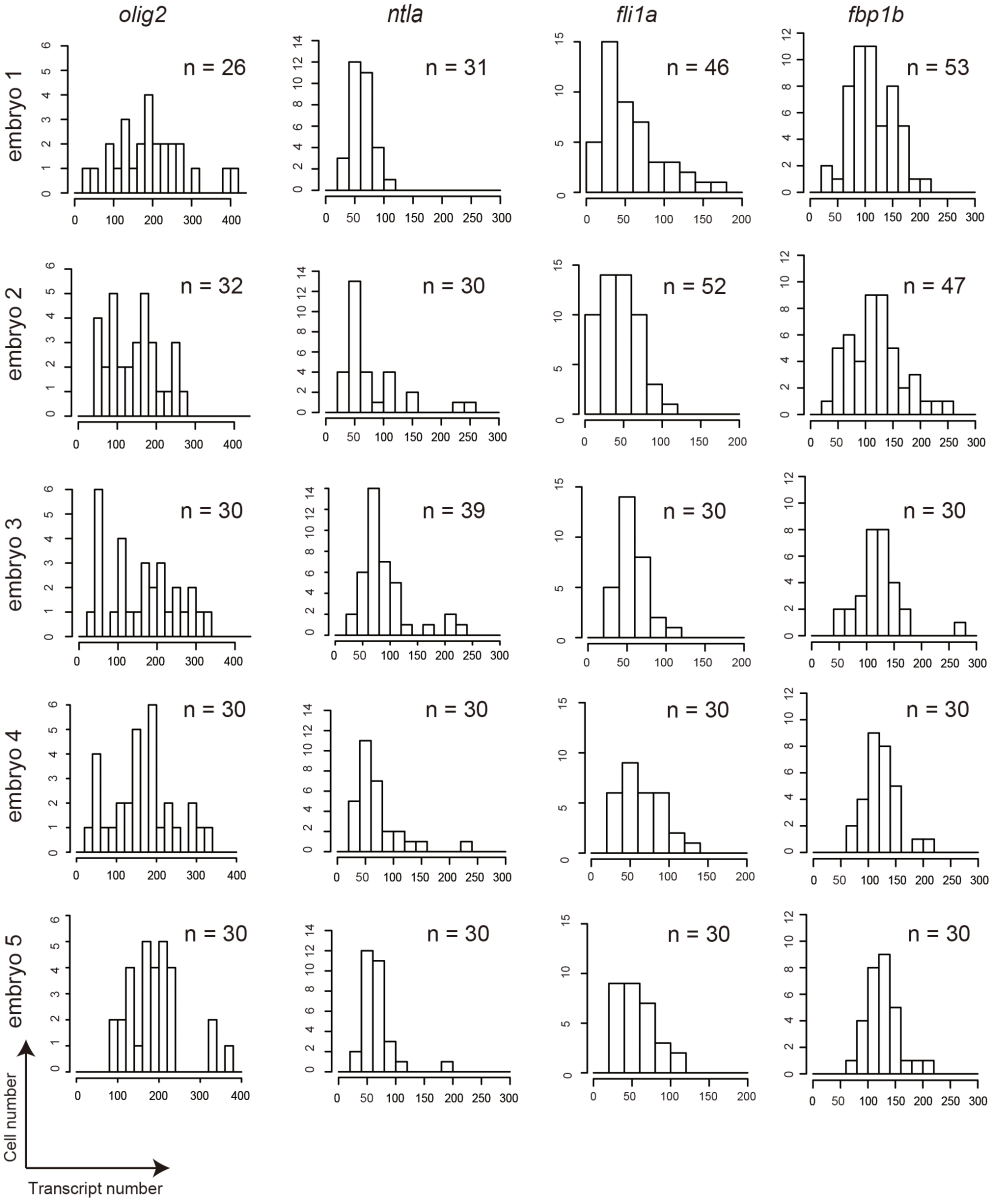
Probability distributions of the transcript number for each gene in individual cells. Histograms of the transcript number for each gene (*olig2*, *ntla*, *fli1a*, *fbp1b*) in individual cells from five different embryos (embryos 1, 2, 3, 4, 5). A total number (n) of cells counted for each gene are indicated in each histogram.

Finally, the probability distributions of the transcript number for each gene in individual cells were analyzed using Kolmogorov-Smirnov test (Supplementary Table 2) to determine whether the stochastic gene expression for different genes are under distinct regulatory mechanisms. The transcript number for each gene was compiled from the analysis of five embryos and the number of fluorescent dots was counted from 26–53 cells per embryo (Figure 4). The analysis found that the probability distribution of *ntla* transcripts uniquely fits to logistic distribution, but not to any others (Supplementary Table S2). In contrast, the probability distributions of others (*olig2*, *fli1a*, *fbp1b*) fit to all except Poisson distributions (Supplementary Table S2). These results suggest that the stochastic *ntla* gene expression is under a unique regulatory mechanism.

## Discussion

Herein, we report an smFISH protocol that is applicable to whole-mount zebrafish embryo. The protocol can quantify the transcript levels for genes that are expressed in specific cell types (*olig2*, *neurog1*, *ntla*, *loxl2b*, *fli1a*, *kdrl*, *fbp1b*, *prox1a*) and those that are ubiquitously expressed (*gapdh*, *sdha*). It also detects genes that are expressed at lower levels (<50 copies per cell) (*fli1a* in Figure 4, as compared to *olig2* that is expressed at >150 copies per cell). The smallest target transcript that is detectable in this study is 720 and 1331 bases in length for exogenously (*egfp*) and endogenously (*gapdh*) expressed genes, respectively (Supplementary Table S1). Can our method be used to detect a small RNA such as microRNA in whole-mounted zebrafish embryo? In our method for whole-mount zebrafish embryos and also in the conventional smFISH method for cultured cells, the detection of individual transcript molecules requires about 30 fluorescent probes each hybridizing to tandemly arrayed target sequences, each 20 bases in length and separated by 2 or more nucleotides, on the single transcript molecule. Furthermore, the target sequences must be unique to minimize non-specific hybridization to non-target sequences. Thus, the theoretical minimum length of the individual transcript detectable using smFISH is approximately 700 bases. It was previously reported that certain microRNA species can be detected in cultured cells using the combination of locked nucleic acid (LNA) probes with enzyme-labeled fluorescence^30^. Whether such a unique method combining LNA with enzymatic signal amplification system is applicable to whole-mount embryos remains a challenge for future investigation.

The method can also quantify two transcripts simultaneously in the same single cell or two neighboring cells in the same embryo (Figure 3a, b). While all TAMRA probe sets tested in this study work, Quasar 670 probe sets for some of the genes (*ntla*, *loxl2b*, *fbp1b*, *prox1a*) fail to produce discernible fluorescent signals (Supplementary Table S1). This might be in part due to the fact that TAMRA produces much stronger fluorescent signal (i.e., “brighter”). Fluorescent signals using the probe sets for two ubiquitously expressed genes (*gapdh*, *sdha*) are detected in some cells but not in others (Supplementary Table S1). This result suggests that these genes that are conventionally known as ubiquitously expressed, may in fact show varying levels of expression among different cells and/or cell types, thus only the cells where the expression level is above the detectable threshold can be identified.

We provide several lines of evidence indicating that each fluorescent dot specifically represents a single target transcript molecule: 1) Specific fluorescent signals are detected in HO and HE embryos (Figure 1a, left and middle columns, respectively), but not at all in the wild type embryos (Figure 1a, right column); 2) The number of fluorescent dots in HO increases linearly according to the increase of the transcript level (1.6-fold increase as determined by qRT-PCR) in HO as compared to that in HE (Figure 1b, c); 3) Specific fluorescent dots are detected in the embryos to which *in vitro* transcribed *egfp* RNA is injected, but not in the uninjected embryo (Figure 1d, e); 4) The number of fluorescent dots in the embryos to which *in vitro* transcribed *egfp* RNA is injected increases linearly according to the increasing amounts of the injected *egfp* RNA present in the individual cells (Figure 1d, e); 5) Analysis of the distribution of dot intensity obtained using this whole-mount smFISH protocol (Supplementary Figure S3) indicates that nearly 90%–100% of the dots show a very similar fluorescent intensity (0.25–0.35). Taken all together, these results strongly indicate that each fluorescent dot signal is specific to a single transcript molecule.

The detection efficiency of our method was tested by applying probes with alternating fluorescent dyes (TAMRA and Quasar 670) (Supplementary Figure S4). Each probe is targeted to non-overlapping tandemly arrayed 19 or 20-nucleotide base sequences within *olig2* transcript and is alternately labeled by TAMRA and Quasar 670. Hybridization of the whole-mount embryos with the mixture of TAMRA and Quasar 670 labeled probes produced typical fluorescent dot signals with both probes (Supplementary Figure S4a). The expected number (ca. 53–143) of fluorescent dot signals for this transcript (*olig2*) is found with both fluorescent dyes in individual cells (Supplementary Figure S4a, b). Approximately 80% of the dots detected by one channel are also found by the other (Supplementary Figure S4b). This detection efficiency is comparable to that previously reported for the conventional smFISH applied to *Drosophila* embryos^31^.

The most labor-intensive step in this whole-mount smFISH protocol is to define cell borders. We modified the previously published MATLAB program^32^ to make the image overlaying and fluorescent dot counting step semi-automated, however, defining the borders were performed manually for individual cells. This latter part is time-consuming, and also requires DIC images of whole-mounted embryos. Therefore, the future development of computational and fully-automated tools to define cell borders (e.g., by combining with membrane label of a fluorescent protein) is expected to allow more robust ways for counting the dots in individual cells.

Recently, application of single cell qRT-PCR or RNA-seq analyses to oocyte/early neuroblast^8^ or lung epithelium development^11^, respectively, allowed reconstruction of lineage hierarchy at single cell resolution. These are powerful tools, however, they do not provide single-cell-level-spatial resolution – i.e. incapable of distinguishing neighboring cells of the identical type.

This smFISH method together with single cell qRT-PCR or RNA-seq analyses with sorted cells from zebrafish embryo provides a quantitative tool for unveiling regulatory mechanism for developmental, physiological and disease processes with the single cell resolution. Furthermore, this smFISH method for whole-mount zebrafish embryos may be adapted for the use of other vertebrate model organisms such as frog, chicken and mouse.

## Methods

### Zebrafish

Fertilized eggs were collected and raised in Egg raising buffer (0.06% artificial marine salt supplemented with 0.0002% methylene blue) at 28°C until 24 hpf. To prevent pigmentation, the medium was changed to 1/3 Ringer's medium (1.67 mM HEPES, 38.7 mM NaCl, 0.97 mM KCl, 0.60 mM CaCl_2_, pH 7.2) containing 0.001% phenylthiourea (PTU) (Sigma). The transgenic lines, *Tg(olig2:egfp)* (kindly provided by Dr. Appel) and *Tg(fli1:egfp)* (obtained from Zebrafish International Resource Center), were as previously described^33^,^34^. Animal experimentation in this study was carried out in accordance with animal protocols approved by the Animal Care and Use Committee of Advanced Telecommunications Research Institute International (Permit Number: A1403).

### *In vitro* transcription

The plasmid construct containing *egfp* cDNA (720 bp) and SV40 polyadenylation signal was linearized and *in vitro* transcription of the sense RNA was performed using SP6 RNA polymerase. The *in vitro* transcribed *egfp* RNA was precipitated by LiCl and quantified by NanoDrop 2000. Approximately 1 nl of the known concentrations of *egfp* RNA (2 ng/μl, 10 ng/μl, 50 ng/μl in dH_2_O) was injected into each one-cell stage embryo. Uninjected and RNA injected embryos (12 hpf) were subjected to whole-mount smFISH.

### Probe design and synthesis

The smFISH probes were designed using the probe design tool at http://www.biosearchtech.com/stellarisdesigner/. We designed 29–48 probes (the probe length was 19 or 20 bases for *olig2* or all the other genes, respectively) per gene, depending on the gene sequence length (Supplementary Data). The designed probes are synthesized and labeled with Quasar 570 (Cy3 replacement), TAMRA or Quasar 670 (Cy5 replacement) at the 3′ ends at Life Technologies (*egfp*) or Biosearch Technologies (*gapdh, sdha, olig2, neurog1*, *ntla, loxl2b*, *fli1a, kdrl*, *fbp1b*, and, *prox1a*). For the double-staining experiment (Supplementary Figure S4), a total of 54 probes, each targeted to non-overlapping nucleotide sequences (19 or 20-bases in length) of *olig2* transcript and alternately labeled by TAMRA and Quasar 670, were designed and synthesized by Biosearch Technologies. See Supplementary information for probe sequences.

### Whole-mount smFISH

Whole-mount smFISH protocol for zebrafish embryo was developed by adapting smFISH for mouse cultured cells and *Drosophila melanogaster*^16^,^35^, and standard whole-mount *in situ* hybridization for zebrafish embryo^36^. Embryos were dechorionated with tweezers if they were not hatched prior to fixation. Approximately 10 embryos were treated in 1.5 ml microcentrifuge tube. They were first briefly washed with 1 ml PBS once and then fixed in 1 ml 4% PFA at 4°C overnight. Embryos were then briefly washed with 1 ml ice-cold PBS once and soaked in 1 ml cold methanol. The embryos were incubated at −30°C for 30 min. For rehydration, embryos were washed sequentially with 1 ml 50% methanol/50% PBSTw (1× PBS and 0.1% Tween-20) and then 1 ml 100% PBSTw at room temperature for 5 minutes each. Although not essential, in some cases, the head was cut and separated from the rest of the body using a razor blade for easier mounting of the brain tissue in dorsoventral orientation for the later microscopy work. In this case, the head tissue separated from the rest of the body was then briefly washed with PBSTw prior to the prehybridization step. While the separation of the head from the rest of the body could be performed after the hybridization step for the imaging work, such procedure could cause significant photobleaching as the embryos hybridized with fluorescent probes become exposed to light under the dissection microscope while the head tissue is removed. After the fixation, rehydration and washing, the embryos were incubated in 300 μl pre-warmed prehybridization buffer (10% formamide, 2× SSC, 0.1% Triton X-100, 0.02% BSA, and, 2 mM Ribonucleoside Vanadyl Complex (New England Biolabs)) at 30°C for 5 minutes. The probe stock solution (25 μM) was diluted to 1:100 in 100 μl hybridization buffer (prehybridization buffer + 10% dextran sulfate) (i.e., the final concentration of the probe set is 250 nM). For the double-staining experiments, 1 μl (25 μM) of each probe set was diluted in 100 μl hybridization buffer. The embryos were soaked in 100 μl hybridization mix in 1.5 ml microcentrifuge tube and incubated at 30°C overnight in dark. On the following day, embryos were washed twice with 1 ml wash solution (10% formamide, 2 × SSC, and, 0.1% Triton X-100) at 30°C for 30 minutes each, and then briefly washed with 1 ml 2 × SSC. Embryos were mounted on slide glass with ProLong Gold antifade reagent with or without DAPI (Life Technologies). For 12 hpf embryos, yolk was removed by tweezers mechanically and the deyolked embryos were flat-mounted on slide glass.

### Microscopy

Samples were observed using 100× alpha Plan-Apochromat NA 1.46 oil immersion objective lens equipped on LSM710 (Carl Zeiss) and 60× Plan Apo NA 1.40 oil immersion objective lens equipped on A1 Plus (Nikon). For DIC imaging, 10× Plan Apo λ NA 0.45 objective lens was used. We acquired three-dimensional stacks with 2048 × 2048 pixels and z-spacing was 0.3 μm for LSM710 and 0.2 μm for A1 Plus. The voxel size was 0.04 μm × 0.04 μm × 0.3 μm for LSM710 and 0.10 μm × 0.10 μm × 0.2 μm for A1 Plus. The excitation lasers were 488 nm for EGFP, 514 nm for Quasar 570, 561 nm for TAMRA, and 630 nm for Quasar 670.

### Image analysis

The acquired image data were converted from. lsm (LSM710) or. nd2 (A1 Plus) to. tif using Imaris. The spots were semi-automatically counted using the previously published program written in MATLAB (Mathworks)^32^ with the following modifications. The region of interest (ROI) setting for specifying cell borders was modified (Supplementary Figure S5). First, the TIFF-format slice images were imported as stacked images. The stacked images were then converted to double, and then filtered with a three-dimensional (3D) Laplacian of Gaussian filter to enhance the dot-like signals, each dot representing a transcript. The borders of individual cells were drawn manually using freehand ROI creating function (imfreehand) on each slice by referring to DIC images, and the slices with the cell border were stacked and the three-dimensional cell border image was generated. The 3D cell border image was then applied to the filtered 3D image. These steps were repeated for each cell. The pixel values for the outside of the cell border were set at 0 for masking. The number of connected objects in the filtered 3D images was counted by assigning 100 distinct thresholds depending on the intensities of the dot-like signals to count the transcript numbers over a certain threshold. The optimal threshold for each 3D image was manually selected as the one that yields the dot-like signals that match to the original dot-like signals in the filtered 3D images.

### Measurement of fluorescent dot intensity

The threshold of 0.25 was applied to all the equally filtered raw images. Fluorescence intensity of each dot was determined by measuring the pixel values for each fluorescent dot and the mean was determined. All fluorescent dots in each cell were counted and compiled for the statistical analysis.

### Quantitative RT-PCR

Total RNA was extracted from 12 hpf 20 homozygous and hemizygous *Tg(olig2:egfp)* embryos using RNeasy Mini Kit (Qiagen). For the *in vitro* transcribed *egfp* RNA injection experiments, 4 RNA-injected 12 hpf embryos of which EGFP expression was confirmed by observation or 4 mock-injected control 12 hpf embryos were harvested for RNA purification. The 500 ng RNA was used as a template for first stand cDNA synthesis using Transcriptor First Strand cDNA Synthesis Kit (Roche) and anchored-oligo(dT)_18_ primer. The quality and amount of RNA were analyzed by NanoDrop 2000 (Thermo Scientific). The primers of *egfp* were 5′- GCCGACAAGCAGAAGAACGG -3′ and 5′- AGGTAGTGGTTGTCGGGCAG -3′. The primers of *rpl13a* were 5′- TCTGGAGGACTGTAAGAGGTATGC -3′ and 5′- AGCGCACAATCTTGAGAGCAG -3′. Reaction mixes (10 μl) were assembled in 384 well plate as follows: 5 μl Master Mix of LightCycler 480 SYBR Green I Master (Roche), 0.5 μl of forward primer (10 μM), 0.5 μl of reverse primer (10 μM), and 4 μl of template cDNA. Templates were 1:40 diluted cDNA samples. The qRT-PCR reactions were carried out using LightCycler 480 instrument II (Roche). The total of 35 cycles were performed with the each cycle consisting of pre-incubation at 95°C for 10 minutes, denaturation at 95°C for 10 seconds, annealing at 60°C for 10 seconds, extension at 72°C for 10 seconds, then back to denaturation. The ΔΔCt method was used for the calculation of the expression level.

### One-sample Kolmogorov-Smirnov test

The fitness of transcript distributions of *olig2*, *ntla*, *fli1a*, and, *fbp1b* to reference distributions (normal distribution, gamma distribution, logistic distribution, Weibull distribution, and, Poisson distribution) were tested with one-sample Kolmogorov-Smirnov test by using ks.test function in R. The parameters of reference distributions were calculated with maximum-likelihood fitting by using fitdistr function in R to each transcript distributions.

## Author Contributions

Y.O. performed all experiments. T.N.S. conceived the project idea, designed and supervised the project. Y.O. and T.N.S. wrote the manuscript.

## Supplementary Material

Supplementary Information Supplementary materials
