# Supplementary material for: Whole-mount single molecule FISH method for zebrafish embryo
Source: Sci Rep. 2015 Feb 25;5:8571. doi: 10.1038/srep08571 (PMC4339797; doi:10.1038/srep08571)
Supplement: Supplementary Information — Supplementary materials [file srep08571-s1.pdf]

**Supplementary materials to:**

# Whole-mount single molecule FISH method for zebrafish embryo

Yuma Oka and Thomas N. Sato

**Supplementary Figure S1. Whole-mount smFISH with and without the methanol pretreatment.** Filtered and threshold applied single plane confocal images of whole-mount smFISH for *gapdh* in neural plate region of 12 hpf *Tg(olig2:egfp)* embryo. Shown are the results with Quasar 570 conjugated probes.

**Supplementary Figure S2. Histograms of the *egfp* transcript number in individual cells of homozygous and hemizygous transgenic zebrafish embryos.** The number of fluorescent dots are counted for each of the indicated number (n) of cells in each embryo and plotted.

**Supplementary Figure S3. Distribution of the fluorescence intensity of individual transcript dot.** Mean pixel values of each fluorescent dot in each cell (Cell #1, #2, #3, #4) are determined and plotted for each cell. Histograms are shown on the left and the plots of mean intensity value for each dot are shown on the right.

**Supplementary Figure S4. Detection of *olig2* transcripts by probes with alternating fluorescent dyes.** (a) Images of individual cells taken by two different channels. Dots with TAMRA (green) and Quasar 670 (red) probes are shown. Yellow dots in the merged image indicate the transcripts co-stained by probes with both colors. Individual cells are enclosed by solid while-lines. Scale bars: 4 μm. (b) Histograms indicating the numbers of fluorescent dots in five individual cells (#1, #2, #3, #4, #5). The numbers of dots detected by TAMRA (green bars), Quasar 670 (red bars) and both (yellow bars) are normalized to that detected by TAMRA in each cell and shown as "relative transcript level". Approximately 80% of the dots detected by one fluorescent dye are also positive by the other.

**Supplementary Figure S5. Schematic diagram for counting the number of dots in individual cells.** See the Methods section of the description.

**Supplementary Table S1. Summary of whole-mount smFISH for 10 genes.** The result of whole-mount smFISH protocol for ten different genes examined in this study.

**Supplementary Table S2. Kolmogorov-Smirnov tests for probability distribution of each gene expression.** For each transcript distribution, the equality to reference probability distributions (normal distribution, gamma distribution, logistic distribution, Weibull distribution,

and Poisson distribution) is examined with one-sample Kolmogorov-Smirnov test and the results are shown as P-values.

**Supplementary Data. Nucleotide sequences of the probes for the genes studied in this report.**

Supplementary Figure S1

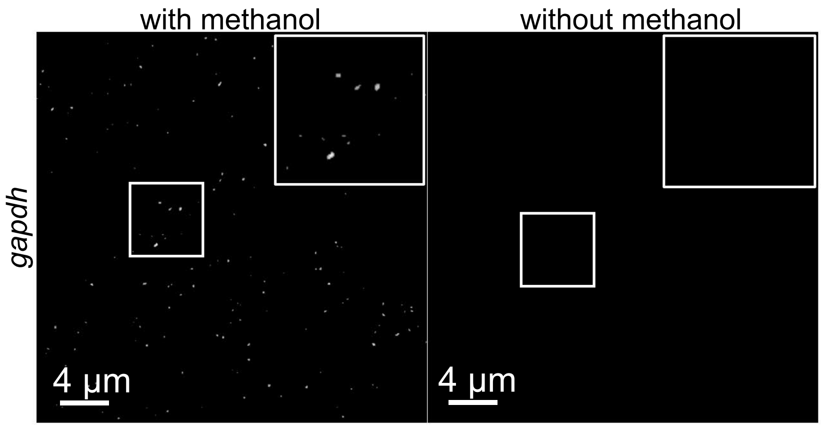

Supplementary Figure S2

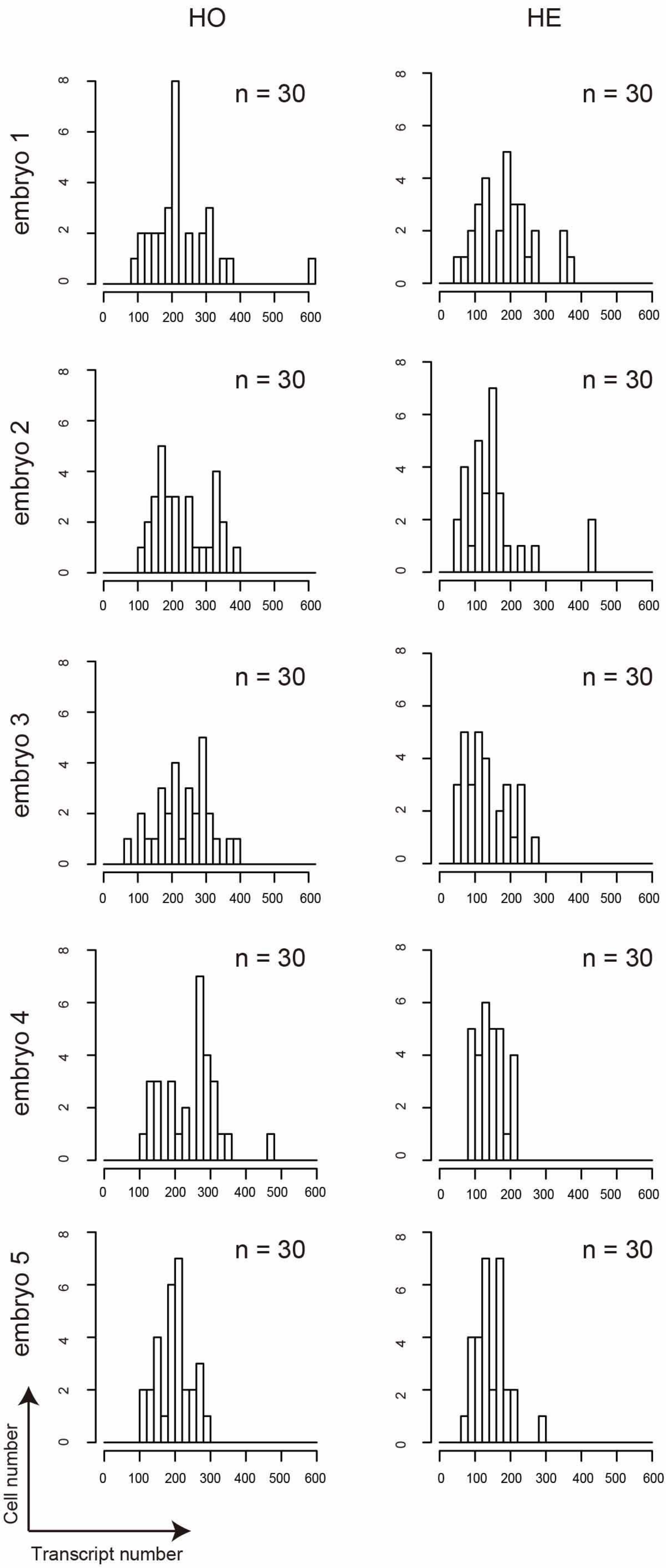

Supplementary Figure S3

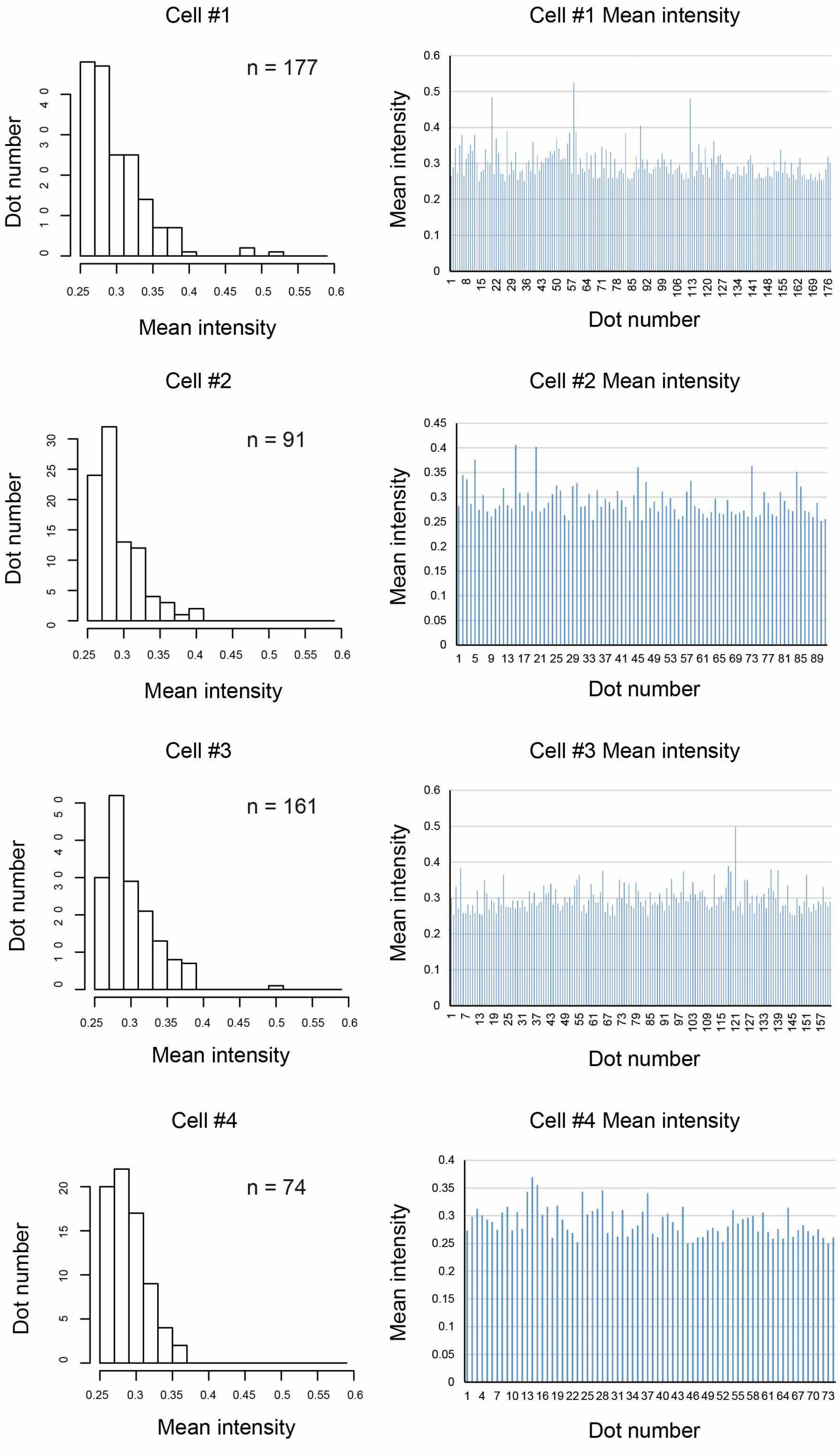

Supplementary Figure S4

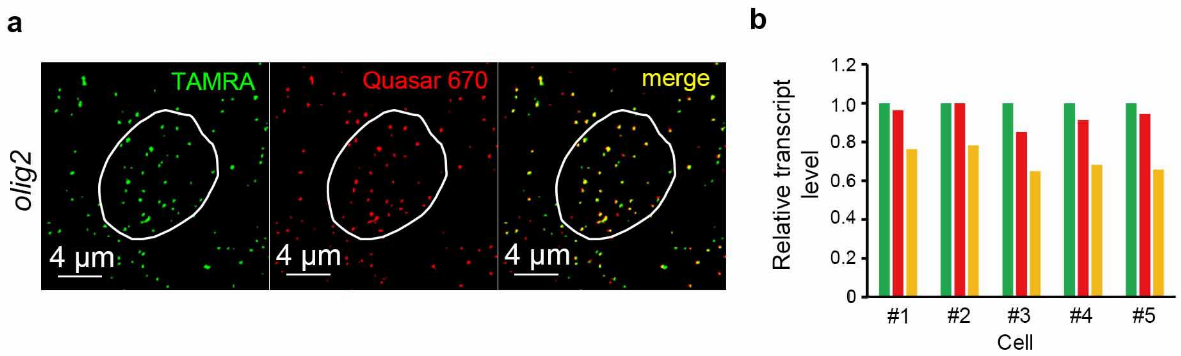

Supplementary Figure S5

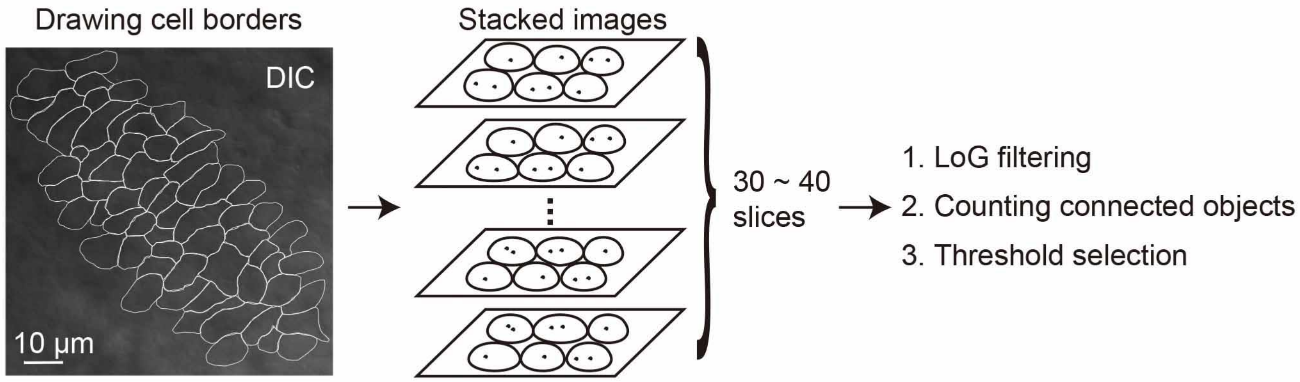

Supplementary Table-S1

| Target gene | Transcript length (bps) | Cell-type | Stage | Fluorescent dye | |
|---|---|---|---|---|---|
| | | | | TAMRA | Quasar 670 |
| *olig2* | 1684 | pMN in proneural domain | 12 hpf | + | + |
| *neurog1* | 1394 | pMN in proneural domain | 12 hpf | + | + |
| *ntla* | 2211 | vacuolar cells in notochord | 1 dpf | + | - |
| *loxl2b* | 2894 | vacuolar cells in notochord | 1 dpf | + | - |
| *fli1a* | 2973 | endothelial cells in brain | 2 dpf | + | + |
| *kdrl* | 5389 | endothelial cells in brain | 2 dpf | + | + |
| *fbp1b* | 1409 | hepatocyte in liver | 4 dpf | + | - |
| *prox1a* | 3008 | hepatocyte in liver | 4 dpf | + | - |
| *gapdh* | 1331 | pMN in proneural domain | 12 hpf | - | - |
| | | vacuolar cells in notochord | 1 dpf | + | - |
| | | endothelial cells in brain | 2 dpf | + | + |
| | | hepatocyte in liver | 4 dpf | + | - |
| *sdha* | 2543 | pMN in proneural domain | 12 hpf | + | + |
| | | vacuolar cells in notochord | 1 dpf | + | - |
| | | endothelial cells in brain | 2 dpf | + | + |
| | | hepatocyte in liver | 4 dpf | + | - |

“-”: Not Detectable

Supplementary Table-S2

| Kolmogorov-Smirnov test | P-value | | | |
|---|---|---|---|---|
| | *olig2* | *ntla* | *fli1a* | *fbp1b* |
| normal distribution | 0.9057 | 1.503e-5 | 0.1416 | 0.5185 |
| gamma distribution | 0.2348 | 0.01115 | 0.7452 | 0.6385 |
| logistic distribution | 0.7980 | 0.07904 | 0.548 | 0.9896 |
| Weibull distribution | 0.8213 | 0.001408 | 0.7655 | 0.3251 |
| Poisson distribution | <2.200e-16 | <2.200e-16 | <2.200e-16 | 5.675e-13 |

Supplementary Data

Probe seqence (5'-> 3')

| | egfp | olig2 | neurog1 | ntla |
|---|---|---|---|---|
| 1 | tcctcgcccttgctcaccat | agaaggtctgctggacact | tgctcttaaccctcaatcag | tcgggacttgaggcagacat |
| 2 | gggcaccaccccggtgaaca | aagatcatcgccttcagga | aagataatggcacgcgtctg | ctaaggagatgatccaggcg |
| 3 | cgccgtccagctcgaccagg | ttcttgacggcggacagaa | atcctgcagatagtttgtgt | ttctgaaattcgctctccac |
| 4 | ctgaacttgtggccgtttac | gagacggcgccggagaaac | gagatgcttgaggttttgca | aagtttaatatcccgctcgg |
| 5 | gccctcgccctcgccggaca | gagaatcgctctgcgttga | tgataaccttattggtgggc | cacaactccgcgtcttcaag |
| 6 | tcagcttgccgtaggtggca | gcatatctgcacctcttaa | cggagtatacgatctccatt | ggtgagctctttaaatttgg |
| 7 | gtggtgcagatgaacttcag | cattgagtcctcctcagcg | tagtcacagcttgaggtttc | ccagtcttggtgacaatcat |
| 8 | ccagggcacgggcagcttgc | ctttttggagagcatcttt | atcatccgtgtgcgaaaagg | tgagcacgggaaacattcgt |
| 9 | tcagggtggtcacgagggtg | tcggagagaagtttacggt | tggagacgcaggtggttttc | cattgcattagggtcgagac |
| 10 | ctgaagcactgcacgccgta | gcatgctctgcagctcatt | ttcttcttcacgacgtgcac | caaaatccagcaggaccgag |
| 11 | cttcatgtggtcggggtagc | cgcggctgttgatctttag | ttaaggttgtgcatcctgtt | attcaccgttcacgtatttc |
| 12 | cggacttgaagaagtcgtgc | cgtccatggcgatgttaag | gcttctcaaagcatccaatg | gtggatgtagacgcagctcg |
| 13 | tggacgtagccttcgggcat | cgtgagcgtagggcatgac | tgtgtcgtcaggaaacgcag | aagatacgggtgctttcatc |
| 14 | cttgaagaagatggtgcgct | agagagcttgcgcacagac | gagtctcaattttggtcagc | ttggagagtttgactttgct |
| 15 | gggtcttgtagttgccgtcg | agcagcagagtggctattt | atgtagttgtgagcgaagcg | taacataatctgtcctcctc |
| 16 | ccctcgaacttcacctcggc | taaggatgtagttgcgcgc | atccggatggtctccgaaag | gggttcgtatttgtgcaatg |
| 17 | gatgcggttcaccagggtgt | catctcctccagcgagttg | atgaagacgacgaggatgcc | ccgactttcacgatgtgtat |
| 18 | tgaagtcgatgcccttcagc | cgtagatctcgctcacgag | ggtctgagttgcagtaagac | ctgactgctgatcattttct |
| 19 | cccaggatgttgccgtcctc | atggaaaccggcgtgatgg | tatccaaaatcgtccatggc | gcaataaactgtgtctcagg |
| 20 | gtagttgtactccagcttgt | tgcgtaagagtgccacaac | ctgtacactacgtcggtttg | tctcttcattctgatatgct |
| 21 | tgatatagacgttgtggctg | aatgcgagacagcagcggg | agatgctaggcacgaagttg | tgtttgattttcagagcggt |
| 22 | ccgttcttctgcttgtcggc | atggtgcaccgggtgagag | accgacatgagaacgcttaa | catcgaggaaagctttggca |
| 23 | ggatcttgaagttcaccttg | actgctggtggaagcagag | tacatacttctggagattct | ttgtggtcacttctctcttt |
| 24 | ctgccgtcctcgatgttgtg | cggaaagagatgctgcggt | agtaacagtggtcttacact | cagattgctggttgtcagtg |
| 25 | tggtagtggtcggcgagctg | ctttgaggagtccgtgatg | gttgtgctcttggttctaat | cagccaccgagttgtgaata |
| 26 | gtcgccgatgggggtgttct | ctacacggacaagggatcc | aggcaaacagtgaataccta | cattgaactgaggagggctg |
| 27 | gttgtcgggcagcagcacgg | gaacgtgagacggaggcac | tctcatttgcagactgtcat | tacgaacccgaggagtgaac |
| 28 | cggactgggtgctcaggtag | atgctgacggcgctcatac | cacgctccaaggaatgcaaa | caagctggagtatctctcac |
| 29 | cttctcgttggggtctttgc | ttttgagtcactggtcagc | attttctctaacggggttct | taatggctgggatatggagc |
| 30 | tccagcaggaccatgtgatc | | caatctgccttgctttttaa | tagttattggtggtagtgct |
| 31 | gagtgatcccggcggcggtc | | tgacttttcaccttggacag | agacttccggaagagttgtc |
| 32 | ttgtacagctcgtccatgcc | | tcagctttatcgctctacaa | tgaccagctgtcatgagacg |
| 33 | | | aactgatttttcacgctcgt | tgttggaggtagtgtttgtg |
| 34 | | | ttcagtctattgtcacagcg | tgcaactgaccacagacttg |
| 35 | | | cataaggccagatctttgtc | taatggagcccgatgctgag |
| 36 | | | tttcttcgggtcaaaataca | gcgtaggaactgagatgtca |
| 37 | | | ggatcagtcggacagatgag | ccgagtaggacatcgaagaa |
| 38 | | | catgagagctggttaactgt | gagggagaggacacaggcag |
| 39 | | | agaaaagtggtgggaaagcc | taggcctggatcgtacattg |
| 40 | | | cgtacaaacatgtttgcacc | cgatggagctctcgaactgg |

Probe seqence (5'-> 3')

| | loxl2b | fli1a | kdrl | fbp1b |
|---|---|---|---|---|
| 1 | gatatagaccagagagccag | tccttaatagttccgtccat | gaaggctttgactgaggttt | atcaaatgcacctctgtcag |
| 2 | tcatactgtgcgtagcttag | tcttcactcaccactgacag | aggaagcagttgtggatcag | cttccaagacaaacctggtg |
| 3 | ggctatagcaaaacccaagt | atggaggctcgaacagagac | catgctgaaggtgtgtatct | ctgttcctttggcttttctg |
| 4 | gaagctctggaggagtgtaa | tgtctgtcttgggtaaagga | taccgatgacacatttctgc | gttgagaagggttgtaagct |
| 5 | cagacgtaactgtatcctgg | aatggggttgattttgtgcg | tgtgtatgactagcttgctg | ctttgatggctgtgcacatg |
| 6 | tatgacttgggctgcatgaa | ctgattaatccactcttgct | aagcctccacaaatggatga | cttttctgacagcagtggaa |
| 7 | gatggagaccaggatattgc | atactctcgcttgacgttca | tgggtatgttgttctacagg | tccatagagattggcaatgc |
| 8 | agtggacgttatcgaaccag | actctctggatccgttgatg | ggaccgtgaaacctttttt | taacatttgtgcttccagca |
| 9 | acacaccaatgccattagac | ccaccattttattgcatttc | gatggtagctgtcataaggt | tagctttttcacctggtctc |
| 10 | gacatcctcagaatgtttgc | tagttcatctgagacgcttc | ccattgttagccttttaagt | ctagatcattggacaggaca |
| 11 | tgaatcctgggattctcttg | acatttctcatccatgtagc | attgtaagtggtctctgcag | acacaggatgtgaaggagga |
| 12 | tgttggtaagggtgttgaca | attggtcgtcatgtttggag | ttgatccgctccttatgaaa | ttcatcttcttcagacacca |
| 13 | aggatagggcggatacgaac | tgatccggagaccacagaga | cattgtcacattaggcacga | ctggttctataatgacggct |
| 14 | actcatcatcacagatctgc | ctgaagaccgtactccttaa | ccatctttgtaccacaagac | aacaaaccacgtatttgccc |
| 15 | ccaaacatgccacagatgac | tgaaacatggccgtgtcgat | cacttgttttatggtcaggt | aatgtttgaagagccatcca |
| 16 | ggtattgtatctcttctgtc | agagctcttttccatctgtg | aagatctttggcttcactct | gttccaatagacgctaagca |
| 17 | cgggcaaacatcttgtacac | aagtcgtctttgctcatctt | ctctggtcatatctgaaggt | tttcctttctgtagattgca |
| 18 | cctttccagtacagtttatg | ataaacgctggtgagtctca | ggcatgggaataccaaatgc | caaggcatccttctctgagg |
| 19 | tgtctgaaggctttcttgta | gatgtgagagaaggacctcg | tgtaagattagcactggggt | taacctgcagccacaatgtg |
| 20 | attctccattcttcaagacc | ctactttccctgaggtaatt | cttgctgtttacatccttta | ggtagcactgccatagagag |
| 21 | ccagttgtcatcacagattg | tgagatggcgtgttgtatga | ccagacacattagctgtttt | tgtcctgtggagagaaccag |
| 22 | aatccaagctcacgacacac | agcatcataagacgcatcgt | aagttcattccttgctgtac | ctagcatgaagcagttgact |
| 23 | caagctgacctccagaaaga | tgtgcatgttgttcgaccag | caaaaggctgtgggtgatca | tatgaactcaccgatggcag |
| 24 | ttctcgaagccagaacactg | gagagaccactgttggagag | gtttctggaaacattgggca | ttaatcctcacatcccgatc |
| 25 | gaaggagcactcagtgacag | tgatccgggtttttggacac | tgatgtttaaagcctggcac | actgtagatctttcctttct |
| 26 | caagaacctcaacacgacct | gataaggatctggctgagga | acatcctgattggtcagatt | aattgctgagcgtagccttc |
| 27 | ccccaaacaagagaaccatt | ctgggttagcaagtcgactg | ccatgtgataaagggtgctg | atattctgtgacatctgggt |
| 28 | actcataaccactgcatctg | tcatcttgaactctccgttg | tcattgctagcacgacattc | ctgggaatttcttcttttgc |
| 29 | acagtgggagagagacatct | cgtagttcatgttgggtttg | taatgaccgcactggttttt | ccataaggtgaactgccatc |
| 30 | tgagatattctccgtgatgc | ttgtcgtagtagtagcgcag | gacaccaagatgatgacctc | ctaccatagaccccacatag |
| 31 | cagtctctgagcaggaaact | cgtgcactttggtcatgatg | tcaggatccatgatgatgga | tagactagggtcctgtgcac |
| 32 | acatgaacataggacggtcc | agtcgaacttgtaggcgtag | caacttttccaaatgctcca | agggtacaggaaaattcctc |
| 33 | ttctcctcataagcacactg | gtacttgtacatggtggact | gtcaatgccaaatgcagagg | ccttgggactcttgacatta |
| 34 | gatctgggaggagaaacgga | cgtgatagctaggcacgtac | actttcagcattttcacagc | tcatacaacagccttagctt |
| 35 | aagtcagattgtccgttgtt | acgaagttgaccttctgctg | agacattaaggctctccatt | atgatgaaggccatggggtt |
| 36 | gttctctctggagattttgg | cattgacggagggtgaggag | tgatgcccaatatggatcag | aacgttcatggctccagttg |
| 37 | ctgtgacagtcatgccaaac | tgggaccgaagaagttggag | tccacgattatcattagggg | ttgattcaggctgaatgtcc |
| 38 | cacttccatgctatgatagt | tgggattggggtaaatacct | ctcttacttctcaggtagtt | agaactacaggaaccctctg |
| 39 | cattggtgctgagtagatca | gtgtgaattggcatggcgtg | gcagtagctcttatcttcaa | tactcctttacatcatcagg |
| 40 | cagacagaagcttgctttgt | aactaccaaggtgtgaaggc | tcaagtcttccaatgtgagc | catttggcatgtttttttgta |
| 41 | caattccttcatcacactct | | cggtggatacattttctcga | |
| 42 | caaagttcgcacactcgtac | | taagggaagtctagcgtctc | |
| 43 | tgtcatgtctgtatgtatcc | | agagagaagatctcccacat | |
| 44 | cccggtttaacatctgtaat | | agaggaggagtactcaggag | |
| 45 | tgtaatccgactcagcaact | | cacactagcctgtagcaaat | |
| 46 | tatacggtgaccatcatacc | | cttcatcgaaggtcttcaca | |
| 47 | taaaggagcctcctatatgg | | gtcttcaggtcatctgaaga | |
| 48 | cagggaaagtgtcttctgtc | | actcagtgagggaacaagcg | |

Probe seqence (5'-> 3')

| | prox1a | gapdh | sdha |
|---|---|---|---|
| 1 | aagagggatgtgctgtcatg | agctcagacattattgcaag | tcgattagtccggtctgatt |
| 2 | tcctatgtcaactcttcttc | ggccaaatccattgattcca | aaaatctttgtcccgaggac |
| 3 | tttgcgcgggtaaaaaccac | ctgaggaccaaacggccaat | atggagaaatgcaactgacg |
| 4 | tggggattcatggcactaag | gcggtgactttaattccctt | tgattggagactccatctga |
| 5 | tgcaccactgaacactcaac | gtactgcaggtcaatgaagg | cgactgcatcaaactcatga |
| 6 | ctcttcaacagcttgcgaag | gccatgagtggagtcatact | ttgcctaacgcagcattaat |
| 7 | atcacagcgtcttcgtaaga | ccatgtgaacttctcccttg | tcaccgtgtcataaaagtgc |
| 8 | ctggaaattaggctcactgc | ctgaccatcaacgatcagtt | gctcggtcatgtagtgaatg |
| 9 | gctgaatggtgaaaggcact | tgcactggaacacagagatg | aacggcataccaaagttctc |
| 10 | tccatctcaaattgggtcat | catgggatctcagcaggctt | gatcttgccatcgtcagtac |
| 11 | aagtgtgacgctgggagaat | gtggactcgaccacgtacag | ttgccaaacttcagactctg |
| 12 | agctgctgaaaactctgctg | ctcaatgctgaggaagactc | catagagagtgtgcagaagc |
| 13 | cgttcttcatgtttctgttc | caccctggatgtgagctgag | agtagctggtatcgtacctc |
| 14 | ggtagaacttctcttggagt | gatggagcagacacaaccac | cagcagatccagagcaaaat |
| 15 | tcatcgttttcagagtcagt | gactcccatcacaaacatgg | gagtgcaatgactcccttac |
| 16 | aacgatctgggacagagtcg | aggggtcatatttgtcctgg | tctgaaacggtggattgatc |
| 17 | aggtcagacatttcgttgtc | cattgctgacaatggtcatg | tggcaatgactgtgttcttt |
| 18 | gtgctcgatccaaaaagtgc | ccaagcagttagtggtgcag | aacaaattcgagatcctggc |
| 19 | tttagcgtttctgccaattg | tgaataactttagccagagg | ccagctccatagatacctgt |
| 20 | gacattgctgtattgagctc | catgaactgtggtcatgaga | cgtaacgctccatgaatctc |
| 21 | caaagaccttcaccacagtg | catcgacagtcttctgtgtg | ggtcatggatcttgaaacca |
| 22 | aaaacctgaggaactgggcg | atcacgccatgccttggcag | aaaaatcatggcggtctcag |
| 23 | tctccattgactgcgaaacg | ccttggcggcaccagtggat | aaactgggataggctctttg |
| 24 | tggcagtatggaagtttgga | ctcaggaatgactttgccca | ccatgttgtagtggactgtg |
| 25 | accaaaacattgcaagcgct | taccagtcagctttccgtta | tataggtgatgacctgtccc |
| 26 | tccaggggattaggaatgat | agcgactggcacacggaaag | aacacaacaagatccagcag |
| 27 | ctctgtttggtcatttgagt | gtgaggtcgacaacggacac | tgtcaatctcagcaatggtc |
| 28 | ccagtctgatctgaagagtt | ccttgatgttggcgtagctg | tcagaggagaaagcttctct |
| 29 | gagggaggactaaaacccat | atgagcggccttcttgacag | ttagcatagcgcatcttgtc |
| 30 | gaggaaggggaaaggatgg | cccagaattcccttcattgg | tctgatctctgaggttctgg |
| 31 | acctaaggggctttggaaag | aaacgactgaatcctctgtg | agaacatctccagtacggaa |
| 32 | aggagaagacctatcctttc | agtgtctccaacaaagtcag | cgacttatagacggactcca |
| 33 | gtggtctctctggaaagatc | ccggcatcaaagattgaaga | atcggtgttccacacaatac |
| 34 | aacgatggtgactgaggtgg | tgagcttgacaaagttgtca | aacatcagattctgcagctc |
| 35 | ccgcattctgactttatgag | tgtagccaaattcgttgtca | gcgcctctactttctttacg |
| 36 | gatgtctgacatgtcctgaa | acattaacaggtcagcaaca | catccacacgatccttgaag |
| 37 | gcgcgtgtagaagaacatca | ggagttactctttggagtgc | ctgcagaggcttggaataat |
| 38 | acatcttcagcatgttggag | aaggaatggtctggcttttc | tcgaaaggcttcttcacttg |
| 39 | gcggttaaacttcacgtctg | gtcacgatggctttagctag | ctgtttcgggatccacatag |
| 40 | tgatcagctgggatgtgatg | gaacgcagcgtctcgtaggt | gggtctgtactccagagtta |
| 41 | aactcgcggaagttgctgaa | acacacagacatctacagag | ctgaagcgtagcctgttaaa |
| 42 | gcaaatttctccatctggat | gacactgacacaaatactgt | atttctgcaagtgctcatgt |
| 43 | ttgtagtgcatgttgagagc | caactacagcaatgcctgtg | acatgaagtctgactggagt |
| 44 | tccggaacctcaaaatcgtt | gtgattacacccaaaatggc | tcatgatttacttgccagcg |
| 45 | tggcattgaaaaactcccgt | cctcatgtaactttattcgg | gtgctacttgacagcgttat |
| 46 | cttcttccaggaaggatcaa | actgcattacagtagccttt | tgcacacaagctgttaagtt |
| 47 | aaaatctctgggacctcact | | caagcgaaaccggatattca |
| 48 | agaagctcctgcagacaatt | | tatttgcagatgaaaggcca |
